# The ODC 3′-Untranslated Region and 5′-Untranslated Region Contain *cis*-Regulatory Elements: Implications for Carcinogenesis

**DOI:** 10.3390/medsci6010002

**Published:** 2017-12-22

**Authors:** Shannon L. Nowotarski, Lisa M. Shantz

**Affiliations:** 1 Division of Science, The Pennsylvania State University Berks Campus, Reading, PA 19610, USA; sln167@psu.edu; 2 Department of Cellular and Molecular Physiology, The Pennsylvania State University College of Medicine, Hershey, PA 17033, USA; lms17@psu.edu

**Keywords:** ornithine decarboxylase, polyamines, untranslated region

## Abstract

It has been hypothesized that both the 3′-untranslated region (3′UTR) and the 5′-untranslated region (5′UTR) of the ornithine decarboxylase (ODC) mRNA influence the expression of the ODC protein. Here, we use luciferase expression constructs to examine the influence of both UTRs in keratinocyte derived cell lines. The ODC 5′UTR or 3′UTR was cloned into the pGL3 control vector upstream or downstream of the luciferase reporter gene, respectively, and luciferase activity was measured in both non-tumorigenic and tumorigenic mouse keratinocyte cell lines. Further analysis of the influence of the 3′UTR on luciferase activity was accomplished through site-directed mutagenesis and distal deletion analysis within this region. Insertion of either the 5′UTR or 3′UTR into a luciferase vector resulted in a decrease in luciferase activity when compared to the control vector. Deletion analysis of the 3′UTR revealed a region between bases 1969 and 2141 that was inhibitory, and mutating residues within that region increased luciferase activity. These data suggest that both the 5′UTR and 3′UTR of ODC contain *cis*-acting regulatory elements that control intracellular ODC protein levels.

## 1. Introduction

Polyamines are small ubiquitously expressed polycations that are essential for normal cell growth and development [1,2]. Their positive charge allows them to bind to DNA, RNA, proteins, and acidic phospholipids [3]. Under normal physiological conditions, the concentration of polyamines is tightly regulated by biosynthetic, catabolic and poorly understood transport mechanisms [4,5]. Ornithine decarboxylase (ODC) is the first and usually rate-limiting enzyme in the polyamine biosynthetic pathway and converts the amino acid ornithine into the diamine putrescine, which is subsequently converted to the higher polyamines spermidine and spermine [6]. Changes in transcription, translation and protein degradation have all been shown to maintain ODC intracellular levels under normal physiological conditions [7,8,9,10]. Recently, our group described the post-transcriptional regulation of ODC through the RNA binding proteins (RBPs) HuR and TTP [11,12].

Post-transcriptional regulation occurs in a variety of transcripts and encompasses mRNA stability and mRNA translation efficiency with both the 3′ and 5′ untranslated regions (UTRs) playing a role in these processes [13,14]. Along with regulation due to secondary structure, 5′UTRs may contain a collection of regulatory elements such as upstream start codons (AUGs), internal open reading frames, and internal ribosome entry sites that affect translation initiation [15]. In addition, the 3′UTR regulates processes such as transcript cleavage, mRNA stability, mRNA localization, and translation [15]. Post-transcriptional regulation can be carried out by RNA binding proteins (RBPs), which can bind to adenosine- and uracil-rich elements (AREs) within either the 5′UTR or 3′UTR [16,17]. Classically, this sequence has been denoted as AUUUA. These sequences behave as *cis*-acting elements and are located in numerous proto-oncogene, cytokine, and transcription factor mRNAs as binding sites for RBPs [18,19,20].

Studies investigating ODC regulation have shown that both the 5′UTR and 3′UTR control ODC mRNA translation. The mammalian ODC 5′UTR is long, consisting of over 300 bases [21,22]. The size of this region, in conjunction with a high G-C content on the 5′ distal end, promotes the formation of secondary structure within the ODC 5′UTR [21,22,23]. In addition, the 5′UTR contains a short internal open reading frame that is located 150 bases upstream of the translational start site [21,22,24]. These features have been found to inhibit ODC translation [8,25]. In studies conducted in ODC-deficient Chinese hamster ovary (CHO) cells expressing a firefly luciferase reporter gene, the inhibitory nature of the ODC 5′UTR was partially released by the addition of the ODC 3′UTR [26]. The goal of the studies described here is to further these previous findings by investigating the influence of the ODC UTRs on luciferase activity in normal keratinocytes, which contain low levels of endogenous ODC, and keratinocyte-derived spindle carcinoma cells with high ODC activity.

Insertion of either the entire mouse ODC 3′UTR or 5′UTR into a luciferase control plasmid resulted in decreased luciferase activity in both C5N keratinocytes and A5 spindle carcinoma cells when compared to cells that contained only the luciferase open reading frame. Deletion analysis identified the region between bases 1969 and 2141 in the ODC 3′UTR as inhibitory. Moreover, mutation of the classical AUUUA sequence within the ODC 3′UTR significantly increased luciferase activity. Overall, these studies identify this AUUUA sequence as a negative regulatory element within the ODC 3′UTR. 

## 2. Materials and Methods

### 2.1. Cell Culture

The C5N and A5 mouse keratinocytes (a generous gift from Dr. Allan Balmain, UCSF, San Francisco, CA, USA) were cultured in Dulbecco’s Modified Eagle’s Medium (DMEM) (Life Technologies, Carlsbad, CA, USA) supplemented with 10% fetal bovine serum (Atlanta Biologicals, Lawrenceville, GA, USA), 1% penicillin streptomycin, and 1% glutamine (Life Technologies). These cells have been described previously [27]. Passages 5–20 were used in the experiments, and experimental results were consistent regardless of passage number. Stock flasks were incubated at 37 °C in a humidified atmosphere of 95% air/5% CO_2_. Cells were passaged one time after thawing and before use.

### 2.2. ODC 3′UTR and 5′UTR Luciferase Assays

**Figure 1:**
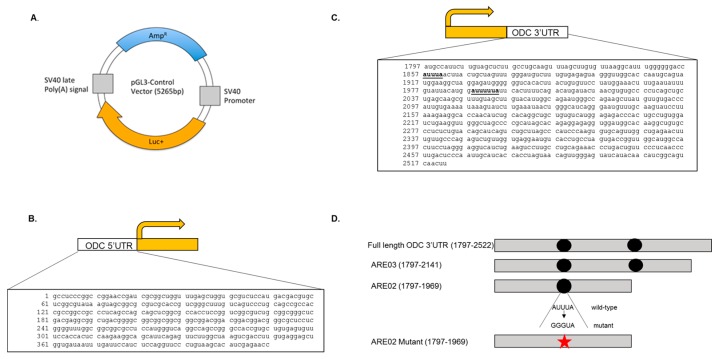
Luciferase constructs used to measure the influence of the ornithine decarboxylase (ODC) untranslated regions (UTRs) on luciferase activity. (**A**) Schematic of the base pGL3 control vector (adapted from Promega). The ODC 5′UTR and 3′UTR sequences were ligated immediately upstream or downstream of the luciferase open reading frame (Luc+), respectively. (**B**) Schematic of the ODC 5′UTR luciferase plasmid (pODC5′UTRLuc) and sequence of the complete ODC 5′UTR that was cloned upstream of the luciferase reporter gene. (**C**) Schematic of the full length ODC 3′UTR luciferase plasmid (pODC3′UTRLuc) and the complete ODC 3′UTR sequence that was cloned downstream of the luciferase reporter gene. Putative adenosine- and uracil-rich elements (AREs) are in bold and underlined. (**D**) Schematic of the full length ODC 3′UTR and distal end truncation constructs used in the luciferase experiments. The full length ODC 3′UTR vector was comprised of bases 1797–2522, the ARE03 vector was comprised of bases 1797–2141, and the ARE02 vectors were comprised of bases 1797–1969. Black circles indicate the location of putative ARE sequences. Star denotes the location of the AUUUA to GGGUA mutation in the ARE02 vector.

The mouse ODC 3′UTR (NM_013614) was cloned into the pGL3 control vector (Promega, Madison, WI, USA) and placed downstream of the firefly luciferase reporter gene while the ODC 5′UTR (NM_013614) was cloned into the pGL3 control vector (Promega) and placed upstream of the firefly luciferase reporter gene. These plasmids are denoted pODC3′UTRLuc and pODC5′UTRLuc respectively. Additional changes in the ODC 3′UTR include a 381 base pair truncation of the distal ODC 3′UTR denoted herein as ARE03 and a 553 base pair truncation of the distal ODC 3′UTR denoted ARE02, cloned into the pGL3 control vector downstream of the firefly luciferase reporter gene (Figure 1). Site-directed mutagenesis was conducted to change the AUUUA sequence present in the ARE02 vector to GGGUA using the Stratagene Quikchange Site-directed Mutagenesis Kit as per the manufacturer’s instructions (Stratagene, La Jolla, CA, USA) (Figure 1). This mutation was introduced to determine whether the putative RBP binding site influenced luciferase activity. The primers used to create the AUUUA to GGGUA mutation were:

5′-GGCATTTGGGGGGACCGGGTAACTTAATTACTGCTAGTTTGG-3′ (sense);
5′-CCAAACTAGCAGTAATTAAGTTACCCGGTCCCCCCAAATGCC-3′ (antisense).

The AUUUA to GGGUA mutation was validated by sequencing. For all luciferase assays, cells were transfected at 70% confluence with 2 μg per plate of vector using the Lipofectamine 2000 transfection reagent as per the manufacturer’s protocol (Life Technologies). Mock transfected cells were treated with Lipofectamine 2000 only. The pRL-SV40 renilla reporter plasmid (Promega) was transfected at 0.2 μg per plate in order to act as a transfection efficiency control. Forty-eight h post-transfection, cells were harvested in 1× Passive Lysis Buffer and assayed using the Dual-Luciferase Kit as per manufacturer’s instructions (Promega). For each sample, the firefly luciferase activity was normalized to the renilla luciferase activity, and the data were expressed as the firefly/renilla ratio. The data were normalized to the pGL3 control.

### 2.3. Statistics

Results are expressed as means ± standard errors (SE) from three to nine samples. Statistical analysis was performed using Student’s unpaired *t*-test on the Graphpad webtool. *p*-values of <0.05 were considered significant.

## 3. Results

### 3.1. The ODC 5′UTR and 3′UTR Decrease Expression of the Luciferase Reporter Gene

**Figure 2:**
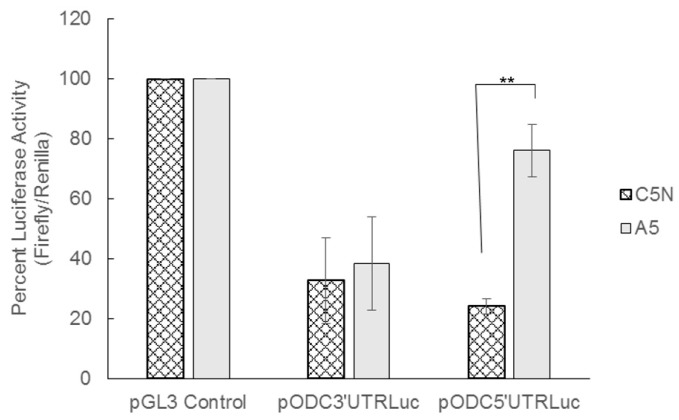
Insertion of the ODC UTRs in the pGL3 control vector causes a decrease in luciferase activity. C5N and A5 cells were transfected with either the pGL3 control vector, pODC3′UTRLuc, or pODC5′UTRLuc. A plasmid containing the renilla luciferase gene was co-transfected into these cells and used as a transfection efficiency control. Luciferase activity was measured 48 h after transfection. Firefly luciferase activity was normalized to renilla luciferase for each sample. The luciferase activity of pGL3 control was set to 100% and the samples from cells transfected with pODC3′UTRLuc and pODC5′UTRLuc are shown as a percentage of the pGL3 control luciferase activity. Values are means ± S.E. (*n* = 9). The difference in luciferase activity between the pGL3 control and both pODC3′UTRLuc and pODC5′UTRLuc were statistically significant for each cell line. ** *p* < 0.005.

To study the influence of the ODC UTRs on luciferase activity, we transfected both normal keratinocyte C5N cells and spindle carcinoma A5 cells with either the parental pGL3 control vector, pODC3′UTRLuc, or pODC5′UTRLuc. We hypothesized that both the 3′UTR and 5′UTR of ODC would greatly reduce the luciferase activity in both cell lines. The insertion of the full-length ODC 3′UTR resulted in an approximate 65% decrease in luciferase activity in both non-tumorigenic C5N and tumorigenic A5 cells (Figure 2).

Insertion of the full-length 5′UTR of ODC resulted in a more dramatic inhibition of luciferase activity in C5N cells when compared to A5 cells. C5N cells showed a reduction in luciferase activity of approximately 80% compared to control, which is similar to previous results in CHO cells [26]. Unexpectedly, luciferase activity in A5 cells transfected with the ODC 5′UTR was reduced by only 20% when compared to the pGL3 control vector (Figure 2). Because the reporter constructs contain identical promoter and enhancing elements, these data suggest the presence of *cis*-acting negative regulatory elements within both the ODC 3′UTR and 5′UTR. Furthermore, the results are consistent with the presence of *trans*-acting factors for the ODC 5′UTR that either enhance expression in the A5 cells or inhibit it in C5N cells, since the ODC 5′UTR sequence is identical in the two cell lines (data not shown). This is in keeping with the higher ODC protein levels observed in A5 cells and would be consistent with an increase in ODC protein synthesis in these cells [12].

### 3.2. The ODC 3′UTR Contains a Negative Regulatory Element between Bases 1969 and 2141

**Figure 3:**
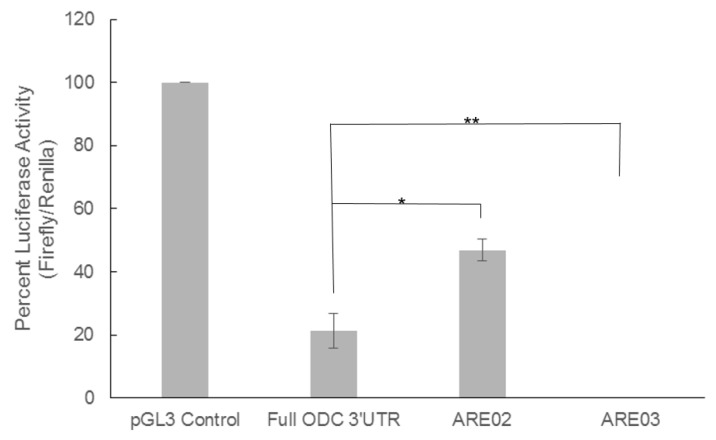
The ODC 3′UTR contains a negative regulatory element between bases 1969 and 2141. The full length ODC 3′UTR as well as two distal end truncations of the ODC 3′UTR were inserted into the pGL3 control vector in order to show the influence of the 3′UTR of ODC on luciferase activity. C5N cells were transfected with either the pGL3 control vector, pODC3′UTRLuc (Full ODC 3′UTR), ARE02 or ARE03. A plasmid containing the renilla luciferase gene was co-transfected into these cells and used as a transfection efficiency control. Luciferase activity was measured 48 h after transfection. Firefly luciferase activity was normalized to renilla luciferase for each sample. The luciferase activity of pGL3 control was set to 100% and the samples from cells transfected with pODC3′UTRLuc (Full ODC 3′UTR), ARE02 and ARE03 are shown as a percentage of the pGL3 control luciferase activity. Values are means ± S.E. (*n* = 6). Differences in luciferase activity between pGL3 control and Full ODC 3′UTR, ARE02 and ARE03 were all statistically significant. Statistics were performed to compare the luciferase activity between Full ODC 3′UTR, ARE02, and ARE03. * *p* < 0.05 and ** *p* < 0.005.

We decided to focus on the influence of the ODC 3′UTR because the effects of the ODC 5′UTR on ODC expression are well-described and because our previous results show that the ODC 3′UTR is important for ODC post-transcriptional regulation through both *cis* and *trans*-acting factors [11,12,26,28]. Given that the luciferase activity was similar between both cell lines, we decided to focus our 3′UTR studies on the C5N cells. To fully understand the impact of the ODC 3′UTR, we measured the luciferase activity in cells that had been transfected with either the full length ODC 3′UTR or two distal end truncation constructs, ARE02 (bases 1797–1969) and ARE03 (bases 1797–2141), both of which contain the classical putative ARE AUUUA (Figure 1 and Figure 3).

While the presence of the full length ODC 3′UTR caused an 80% decrease in luciferase activity compared to control, the ARE02 deletion mutant attenuated this inhibition to 50%. Interestingly, luciferase activity was undetectable in cells transfected with ARE03 (Figure 3), which is 172 bases longer than ARE02. These results indicate that a negative *cis*-regulatory element resides between bases 1969 and 2141. Moreover, the data suggest that the most distal region of the ODC 3′UTR (bases 2141–2522) contains positive regulatory elements, since we see a rescue in luciferase activity when we compare the full length ODC 3′UTR and ARE03.

### 3.3. Mutation of the AUUUA Classical ARE Dramatically Increases Luciferase Activity

**Figure 4:**
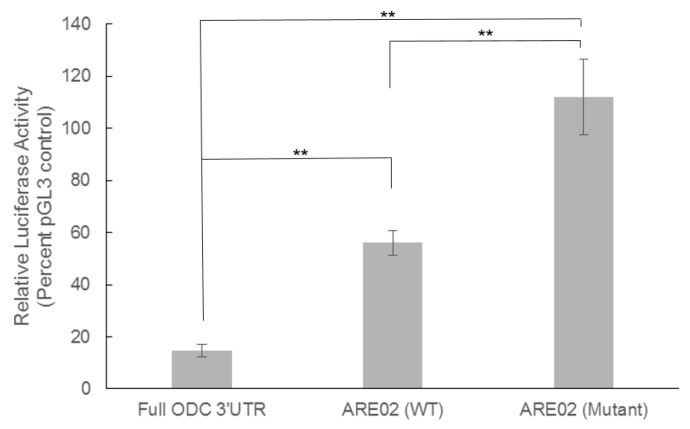
Mutating the AUUUA ARE to GGGUA increases the luciferase activity in non-tumorigenic C5N keratinocytes. Site directed mutagenesis was used to change the AUUUA classical ARE sequence to GGGUA in the ARE02 vector. Sequencing confirmed this 3 base mutation. C5N cells were transfected with either the pGL3 control vector, pODC3′UTRLuc (Full ODC 3′UTR), the ARE02 wild-type plasmid (WT), or the ARE02 mutant plasmid. A plasmid containing the renilla luciferase gene was co-transfected into these cells and used as a transfection efficiency control. Luciferase activity was measured 48 h after transfection. Firefly luciferase activity was normalized to renilla luciferase for each sample. The luciferase activity of pGL3 control was set to 100% and the samples from cells transfected with pODC3′UTRLuc (Full ODC 3′UTR), ARE02 WT, and ARE02 mutant constructs are shown as a percentage of the pGL3 control luciferase activity. Values are means ± S.E. (*n* = 9). ** *p* < 0.005 for all comparisons.

To further elucidate the influence of the AUUUA sequence on luciferase activity we investigated the effect of mutating the AUUUA ARE site on the ODC 3′UTR (Figure 1). These experiments were conducted to verify that the AUUUA site was indeed a *cis*-acting regulatory element on the ODC 3′UTR. Using site-directed mutagenesis we mutated the AUUUA in the ARE02 vector to GGGUA. In C5N cells, the full length ODC 3′UTR exhibited an 85% reduction in luciferase activity compared to pGL3 control, while expression of the ARE02 construct containing wild-type AUUUA resulted in a 45% reduction in luciferase activity. Interestingly, the GGGUA mutant ARE02 construct produced higher luciferase activity than the pGL3 control. These results suggest that the AUUUA sequence acts as an inhibitory *cis*-acting regulatory element within the ODC 3′UTR (Figure 4).

## 4. Discussion

We have previously determined that A5 spindle carcinoma cells have much higher levels of ODC activity and protein than C5N keratinocytes [12]. We therefore decided to investigate the influence of the ODC UTRs in these cell lines using luciferase constructs containing either the wild-type ODC UTR sequences, or a variety of mutations and deletions within the UTRs. In agreement with previous studies, we show that the insertion of the 5′UTR or 3′UTR of ODC into the pGL3 luciferase control vector causes a reduction in the luciferase activity (Figure 2) [26,28]. The level of 3′UTR-influenced luciferase activity inhibition is similar in both C5N keratinocytes and A5 spindle carcinoma cells; however the effect of the 5′UTR on luciferase activity is markedly different between the two cell lines, suggesting that different *trans*-acting factors within these cell lines play a role in ODC regulation. These data fit with our previous work showing that ODC enzyme activity is higher in A5 cells when compared to C5N cells and suggest that a possible mechanism for this difference is altered post-transcriptional control of the ODC mRNA [12]. 

We observed that insertion of the full length ODC 3′UTR dramatically reduced luciferase activity. Moreover, an ODC 3′UTR distal end truncation of 553 bases to create ARE02 mitigates the repression caused by the full length ODC 3′UTR. In contrast, the ARE03 construct (1797–2141) completely ablated the luciferase activity, and addition of the most distal end of the ODC 3′UTR (2141–2522) relieves some of this repression. This is observed when we compare the luciferase activities between ARE03 and the full length ODC 3′UTR (Figure 3). These data can be interpreted two ways, which are not mutually exclusive. First, there are multiple positive and negative regulatory elements along the ODC 3′UTR that may work in concert to regulate the ODC mRNA transcript. Alternately, the secondary structure of the ODC 3′UTR is altered in the truncation constructs, which affects the binding of *trans*-acting factors. We are currently investigating these two possible mechanisms of regulation. Moreover, we are currently investigating the influence of the AUUUUUA sequence on ODC regulation. This non-classical ARE is part of both the full ODC 3′UTR and ARE03 constructs (Figure 1) and may contribute to the inhibitory effect of these two constructs by regulating ODC mRNA stability. 

Mutation of the ARE sequence AUUUA to GGGUA within the ODC 3′UTR resulted in a significant induction of luciferase activity. In fact, the luciferase activity observed in C5N cells that had been transfected with the ARE02 mutant was higher than the luciferase activity in cells transfected with the control vector (Figure 4). These data are in agreement with previous studies using the mouse COX-2 3′UTR, which showed that the removal of the first 60 nucleotides, which contained 7 out of 12 AUUUA sequences, caused an increase in luciferase activity, demonstrating that the AUUUA consensus sequence was inhibitory [29]. Similarly, our results suggest that the AUUUA sequence is an inhibitory element on the ODC 3′UTR.

We previously used Ras-transformed RIE-1 cells, which are characterized by high levels of ODC [30,31] to demonstrate that the RBP HuR bound more strongly to the ARE02 mutant than the wild-type sequence. These findings support our current luciferase activity data as the binding of HuR to the ARE02 mutant would stabilize the mRNA and lead to a higher luciferase activity.

MicroRNAs (MiRs) behave as *trans*-acting factors that are involved in the post-transcriptional regulation of mRNAs by imperfectly binding to a target mRNAs 3′UTR. This binding typically results in accelerated mRNA turnover and a decrease in mRNA translation [32]. MiRNA databases predict that numerous miRs can bind to the ODC 3′UTR, and it has been found that ultraviolet-B (UVB), the primary carcinogen in non-melanoma skin cancer (NMSC), alters the expression of numerous miRs in mice [33]. This has also been demonstrated in human patients who suffer from NMSC [34]. We are currently using our mouse keratinocyte cell models used here as well as a human keratinocyte cell model (HaCaT cells) treated with apoptotic doses of UVB to address the influence of predicted miRs on the post-transcriptional regulation of ODC. We believe these studies will give us better insight into the complex regulation of ODC during the process of skin carcinogenesis.

In conclusion, the studies described here compliment other work investigating regulation by the ODC UTRs [26,30]. We demonstrate using a series of luciferase reporter plasmids that both the ODC 3′UTR and 5′UTR influence luciferase activity, and establish that both positive and negative regulatory elements are contained within the UTRs of ODC. Moreover, we show that a specific site on the ODC 3′UTR, the putative ARE sequence AUUUA, is inhibitory in a keratinocyte model. These studies further our understanding of ODC regulation at the post-transcriptional level. We are currently investigating the mechanisms of ODC 3′UTR and 5′UTR mediated regulation, and how a specific RBP, HuR, is involved in these processes, both in normal keratinocytes and cutaneous carcinomas. We hope to elucidate the binding sequence for this RBP and further investigate the interplay between HuR and other *trans*-acting factors.
